# Targeting neurokinin-3 receptor: a novel anti-angiogenesis strategy for cancer treatment

**DOI:** 10.18632/oncotarget.17250

**Published:** 2017-04-19

**Authors:** Ting Wang, Siwei Chen, Shihui Wang, Liang Shi, Chenggong Wang, Jingxin Zhang, Yanfeng Gao, Guodong Li, Yuanming Qi, Xiuli An, Lixiang Chen

**Affiliations:** ^1^ School of Life Science, Zhengzhou University, Zhengzhou, 450001, P.R. China

**Keywords:** anti-angiogenesis, neurokinin 3 receptor, neurokinin B, antitumor therapy

## Abstract

Angiogenesis is essential for tumor growth and metastasis, controlling angiogenesis is a promising strategy in cancer treatment. However, thus farther severe side effects of anti-angiogenic drugs have been rather demonstrated, stimulating interest in seeking novel targets of anti-angiogenesis. Neurokinin receptors, also known as tachykinin receptors, are usually considered as drug targets due to diverse physiological functions and their tractability. Although Neurokinin B, the selective natural agonist of neurokinin-3 receptor, have been shown to exhibit anti-angiogenesis activity, the effect and mechanism of neurokinin-3 receptor-mediated angiogenesis still remains unclear. In the present study, we demonstrated that [Mephe7]NKB, an analogue of NKB, possess significant anti-angiogenic effect on CAM. Furthermore, by introducing the tumor angiogenesis homing sequence (NGR), we designed and synthesized two novel agonist analogues of NK3R, NK3R-A1 and NK3R-A2. Both of the two analogues exhibit more efficient anti-migration effect on HUVECs by activating NK3R *in vitro*, and showed potent antitumor activities with no significant side effects *in vivo*. Taken together, our results illuminated that NK3R might be a potential novel target for the anti-angiogenesis therapy. Notably, NK3R-A1 might be used as a template for the development of the anti-tumor drugs on the basis of the anti-angiogenesis strategy.

## INTRODUCTION

Since the importance of angiogenesis in tumor development has been recognized over decades [1], interest in the therapeutic potential of anti-angiogenic treatments has continued to grow [2]. The most validated anti-angiogenic strategies act on the VEGF axis, blocking VEGF directly with the neutralizing antibody bevacizumab or the aflibercept (VEGF trap), or indirectly with low-molecular-weight tyrosine kinase VEGF receptor inhibitors (e.g., sunitinib, sorafenib, and pazopanib) [3]. Nonetheless, clinical applications have not got the expected results, only a fraction of cancer patients show benefit as tumors evolve mechanisms of resistance or are refractory toward VEGF (receptor) inhibitors or occur severe side effect, such as bleeding and chemoresistance [4–7]. The limitation of current anti-angiogenic strategies stimulate our interests in finding novel targets for anti-angiogenesis therapy.

Tachykinin receptors, known as neurokinin (NK) receptors, are usually considered therapeutically important due to diverse physiological functions and their tractability as drug targets [8, 9]. Neurokinin B (NKB), the most potent natural agonist ligand for NK3 receptor (NK3R), has been reported to play a vital role in many potentially life threatening disease. Pal et al. demonstrated that NKB could target endothelium via a multi-component mechanism to oppose vascular remodeling and act as an endogenous angiogenesis inhibitor [10]. Thus, we proposed a hypothesis that NK3R might function as a potential novel target for the anti-angiogenesis therapy through the interactions with agonist and analogues of NK3R.

Agonist binding to the receptor is a critical key in initiating signaling and triggering effects, therefore the declaration of structural features of the agonists not only can reveal the molecular basis of receptor activation but also will help in rational design of novel therapeutics. The pharmacology of the NK3R is less well characterized in comparison of NK1 and NK2 receptors. Several agonists derived for the NK3R have been obtained by modification of the primary structure of NKB [11]. [MePhe^7^]NKB, a classical synthetic agonist, has been used extensively to study the role of NK3R and [Gly^6^]NKB[3–10] has been used to facilitate the study of putative NK3R antagonists [12, 13]. Studies with chimeric NK1/NK3 receptors have identified that the carboxyl terminal domain, part of the third extracellular loop and the seventh transmembrane region of the NK3R, is important for NKB binding [14]. Therefore, in order to maintain maximal binding stability and reinforce the affinity with the NK3R, we kept the NKB[4–10] motif and replaced Val^7^ with MePhe^7^.

On the other hand, we considered to bring in additional motif to reinforce tumor targeting effect of analogues of NK3R agonist. A critical component of molecular targeting is the identification of a marker, for instance angiogenic or “activated” endothelium. Novel markers of angiogenic endothelium and their ligands have been identified by various phage display experiments in tumor models. NGR (Asn-Gly-Arg) have been shown to home specifically to tumor vessels in tumor xenograft bearing mice [15]. In addition, NGR conjugated peptides showed a greater anti-tumor effect than uncoupled drugs [16, 17]. The vascular address for NGR has been identified as CD13/APN (aminopeptidase N, also named CD13), which was found to be target for anti-cancerous therapy because of its functional association with the growth of different human cancers. APN could function as the dominating receptor for the NGR peptide motif and this receptor is expressed exclusively on the endothelial cells of tumor vessels in the subsequent research [18–20]. In particular, NGR and Cysteine-asparagine–glycine–arginine-cysteine (CNGRC), which was derived from NGR peptide, have been proven to be useful for delivering cytotoxic drugs, proapoptotic peptides, and tumor necrosis factor-α (TNF) to tumor vasculature.

Here, our result showed that the agonist of NK3R, [MePhe^7^]NKB, could elicit significant anti-angiogenic effect *in vitro* and *in vivo*. Two novel analogues, NK3R-A1 and NK3R-A2, were designed by coupling the targeting anti-tumor motif NGR/CNGRC and the NK3R high affinity binding sequence with a glyciny-glycine (GG) bridge to impart peptide flexibility and minimize potential steric interactions that would prevent binding. Further functional assay indicated that the NK3R analogues exhibit more efficient anti-migration effect than [MePhe^7^]NKB on HUVECs by activating NK3R *in vitro*, and showed no significant side effects on potent antitumor activities *in vivo*. Taken together, our results illuminated that NK3R might be a potential novel target for the anti-angiogenesis therapy. Notably, the modified molecular modeling of the most effective analogue, NK3R-A1, might provide novel insight into the development of anti-tumor drugs on the basis of the anti-angiogenesis strategy.

## RESULTS

### The anti-angiogenic effect mediated by activating NK3R on CAM

**Figure 1:**
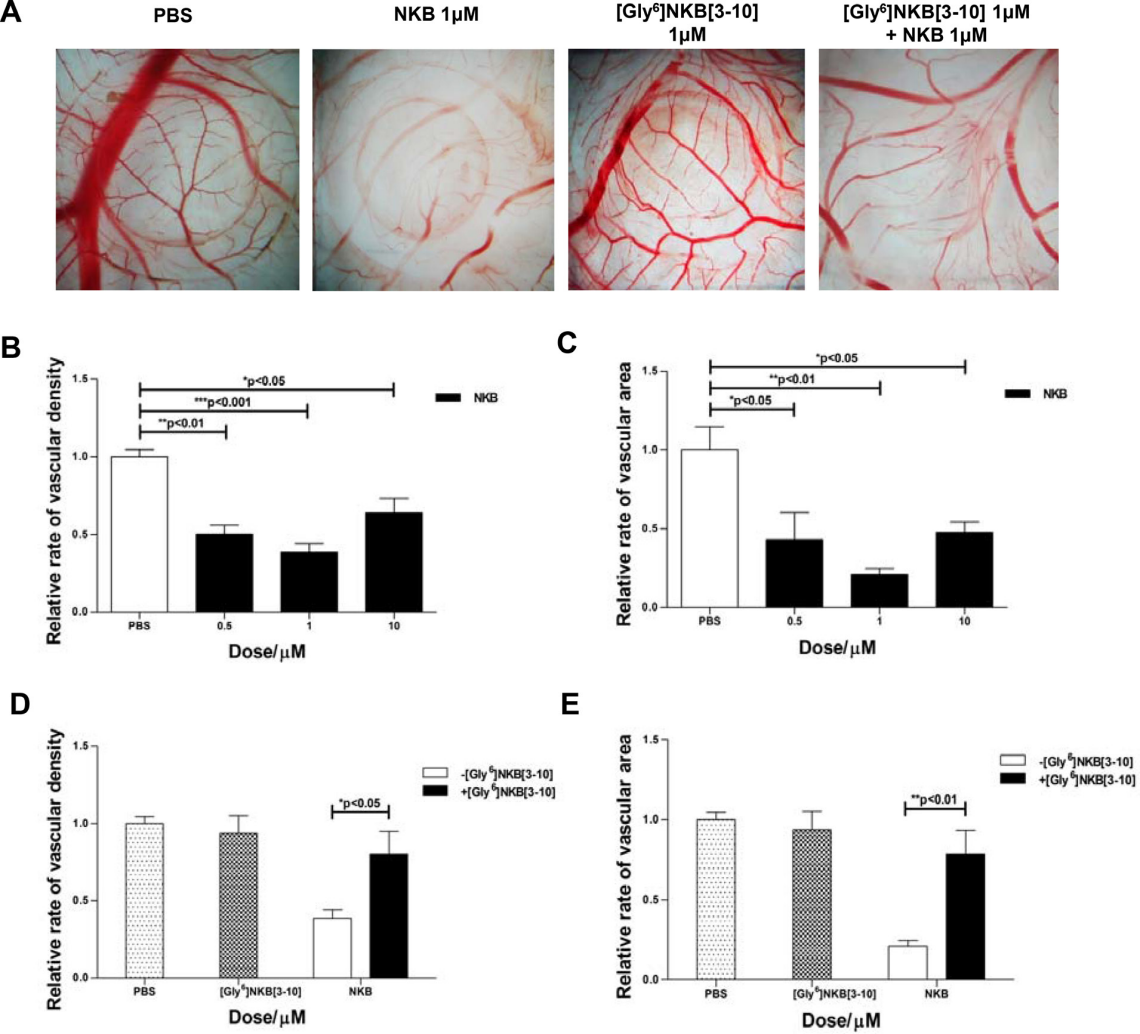
The anti-angiogenic effect of NKB mediated by NK3R on CAM (**A**) Egg CAM was assayed in chick embryos as described in Materials and Methods. PBS, [Gly^6^]NKB[3–10], NKB or a combination of [Gly^6^]NKB[3–10] was applied to the CAM of day 8 chicken embryos. After 48 h, CAMs were dissected out and the representative areas were photographed (*n* = 10). The column charts displayed the relative rate of vascular density (**B**, **D**) and area (**C**, **E**) treated with NKB or co-treated with NKB and [Gly^6^]NKB[3–10]. Data are mean ± SEM from three independent experiments. *p < 0.05; **p < 0.01; ***p < 0.001.

CAM assays were conducted to access whether NKB possess the anti-angiogenic property through its putative receptor, NK3R. Compared to the controls, 1μM of NKB significantly reduced vascular density and bed area to 38.6% and 20.9%, respectively (Figure 1A–1C). [Gly^6^]NKB [3–10], selective antagonist of NK3R, was used for antagonism assay. We found [Gly^6^]NKB [3–10] could dramatically antagonize the effects of NKB to reduce the density of capillary and bed area of the chorioallantois microvessels, while showed no effects alone (Figure 1A, 1D–1E). Our results suggested that NKB possess the anti-angiogenic effect through activating NK3R.

### Selective agonist analogues of NK3R exert anti-angiogenic property on CAM through NK3R

**Figure 2:**
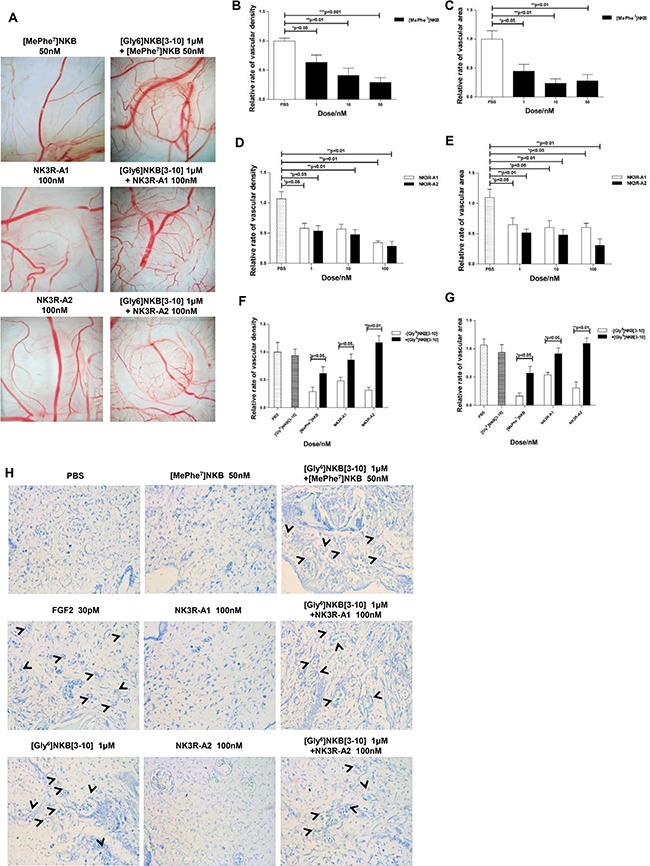
NK3R selective agonist analogues exert anti-angiogenic property on CAM through NK3R (**A**) Chick CAM assay of angiogenesis. [MePhe^7^]NKB, NK3R-A1, NK3R-A2, or a combination of [Gly^6^]NKB[3–10], respectively, was applied to the CAM of day 8 chicken embryos. The representative photos are taken after 48 h (*n* = 10). The columncharts displayed the relative rate of vascular density (**B**, **D**) and area (**C**, **E**) of [MePhe^7^]NKB, NK3R-A1 and NK3R-A2 treated group, respectively, or the relative rate of vascular density (**F**) and area (**G**) co-treated with NK3R selective agonist analogues and [Gly^6^]NKB[3–10], respectively. (**H**) The gelatin sponge-CAM assay of neovascularization at microscopic level on histological sections. Representative photos are shown (400 ×). Data are mean ± SEM from three independent experiments. *p < 0.05; **p < 0.01; ***p < 0.001.

To examine if NK3R could be an effective target for anti-angiogenic strategy, one efficient agonist of NK3R, [MePhe^7^]NKB and two novel designed analogues of NK3R agonist, NK3R-A1 and NK3R-A2 were estimated in CAM assay (Figure 2A). [MePhe^7^]NKB inhibited the expansion of the microvasculature by ~30% at a dose of 50 nM (Figure 2B–2C). Meanwhile, 100 nM of NK3R-A1 and NK3R-A2 remarkably reduced vascular density to 48.4% and 31.6%, and vascular bed area descended to 53.8% and 30.7%, compare to PBS control, respectively (Figure 2D–2E). As we demonstrated earlier, the anti-angiogenic effect of NKB was triggered through NK3R. By pre-treatment with [Gly^6^]NKB[3–10], the anti-angiogenic effects of [MePhe^7^]NKB and two novel designed analogues, were all blocked on CAM in vascular densities and bed areas (Figure 2F–2G). These results indicated that NK3R interaction with all three peptides appear to mediate the blockade of angiogenesis in the CAM assay.

To confirm that our designed peptides indeed possess the property of anti-angiogenesis, especially for neovascularization at microscopic level on histological sections, we further performed gelatin sponge-CAM assay (shown in Figure 2H). Treating with 50 nM [MePhe^7^]NKB, 100 nM NK3R-A1 or100 nM NK3R-A2 respectively, the number of new vessels were decreased dramatically. The antagonist of NK3R, [Gly^6^]NKB[3–10], which did not exhibit obvious anti-angiogenic effect alone, was found to antagonize the anti-angiogenic effects of [MePhe^7^]NKB, NK3R-A1 and NK3R-A2. All the data confirmed that [MePhe^7^]NKB and our novel designed peptides, NK3R-A1 and NK3R-A2, can induce the suppression of neovascularization in the NK3R-dependent manner.

### NK3R-A1, NK3R-A2 and [MePhe^7^]NKB could suppress the migration of HUVECs *in vitro*

We next detected the anti-angiogenic effect of peptides *in vitro*. Given the importance of proliferation and mobility of HUVECs for neovascularization, MTT, wound healing and Transwell migration assays were conducted to further explore the effects of the peptides on HUVECs. In MTT assay, there is no markedly inhibition effects on the proliferation of HUVECs that were treated with [MePhe^7^]NKB, NK3R-A1 and NK3R-A2 for 24 hours (Data not shown).

**Figure 3:**
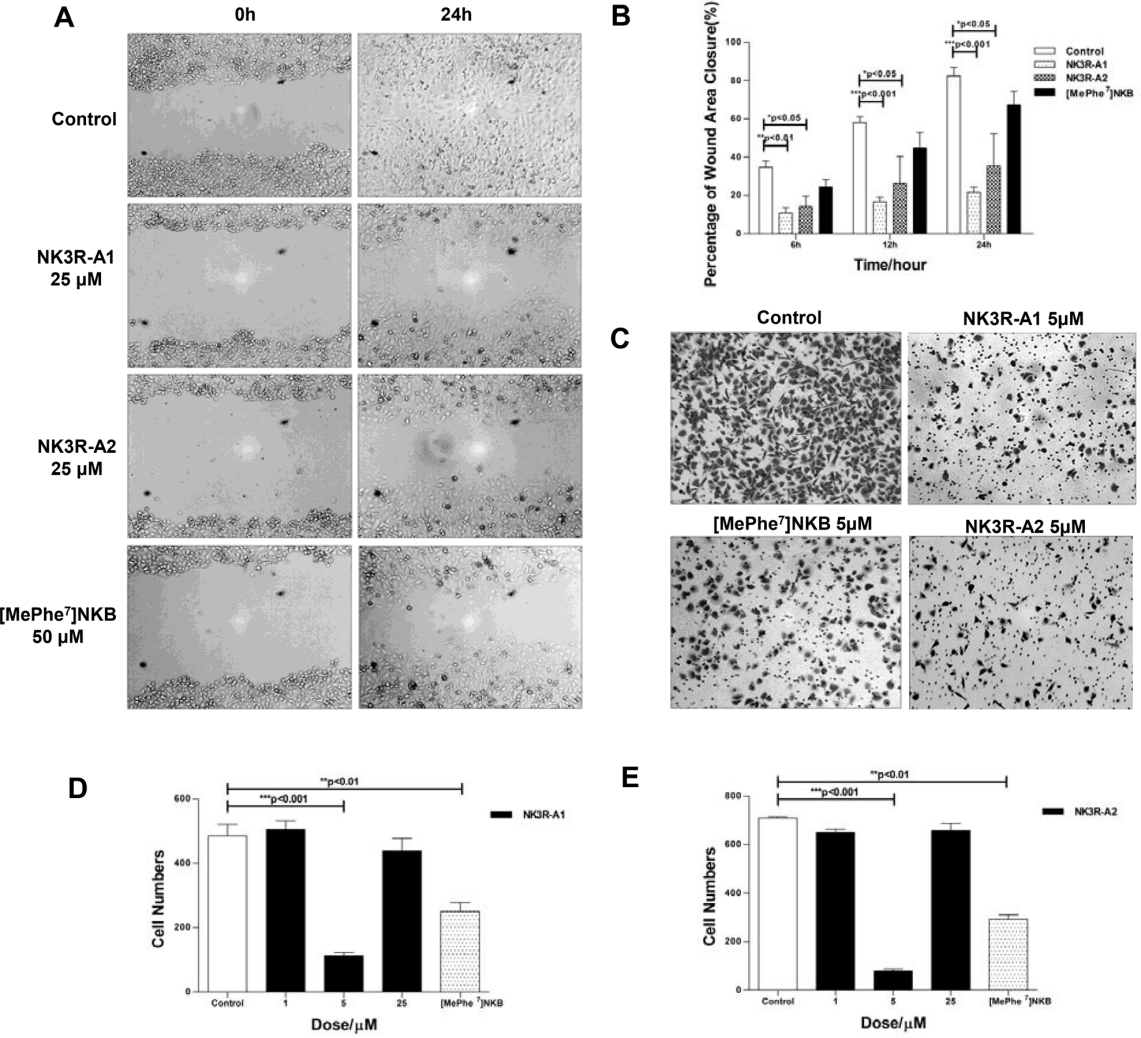
NK3R-A1, NK3R-A2 and [MePhe^7^]NKB could suppress the migration of HUVECs *in vitro* (**A**–**B**) Wound healing assay. HUVECs were treated with PBS, NK3R-A1, NK3R-A2 or [MePhe^7^]NKB. During 24 h healing period, the cells migrating into the wound area were visualized and the digital images were captured for every 6 h, and representative photos were shown (200 ×) (A). (B) The histograms showed the relative width of the wound area within 24 h treated with the optimal concentration of NK3R-A1, NK3R-A2 and [MePhe^7^]NKB, respectively. (**C**–**E**) Transwell migration assay. HUVECs treated with NK3R-A1, NK3R-A2 and [MePhe^7^]NKB were seeded as indicated in the upper chamber. After 12 h incubation, the migrated cells were stained with hematoxylin and photographed (C) and representative images were shown (200 ×). The histograms reflected the number of migrated HUVECs treated with a range of concentration of NK3R-A1 (D) or NK3R-A2 (E) in 12 h. Data are mean ± SEM from three independent experiments. *p < 0.05; **p < 0.01; ***p < 0.001.

In contrast, the peptides exhibit significant affect on the migration ability of HUVECs. Figure 3A–3B shows the results of the migration of HUVECs toward a pseudo-wound inflicted on a monolayer of cells at 0- and 24-hours, respectively. The results revealed that NK3R agonist analogues significantly decrease the closure rate of the wounded area. At around 24 hs post wounding, > 50% of the wounded areas still remain unclosed of the *NK3R-A1 or NK3R-A2 treated group* (Figure 3A–3B), while the wound area of *control* group nearly complete closed.

In parallel, anti-migration effects of peptides were further demonstrated in Transwell migration assay. At 12 hours after seeding, the numbers of migrated HUVECs of NK3R-A1 or NK3R-A2 (5 μM) treated group were 4- and 9-fold less than the control group, respectively (Figure 3C–3E). All the results elucidated that NK3R-A1, NK3R-A2 and [MePhe^7^]NKB possessed the property of anti-migration *in vitro*, and two NK3R agonists analogues showed profound inhibitory effect than that of [MePhe^7^]NKB.

### NK3R antagonist [Gly^6^]NKB[3–10] could antagonize the anti-angiogenic effect induced by the analogues *in vitro*

**Figure 4:**
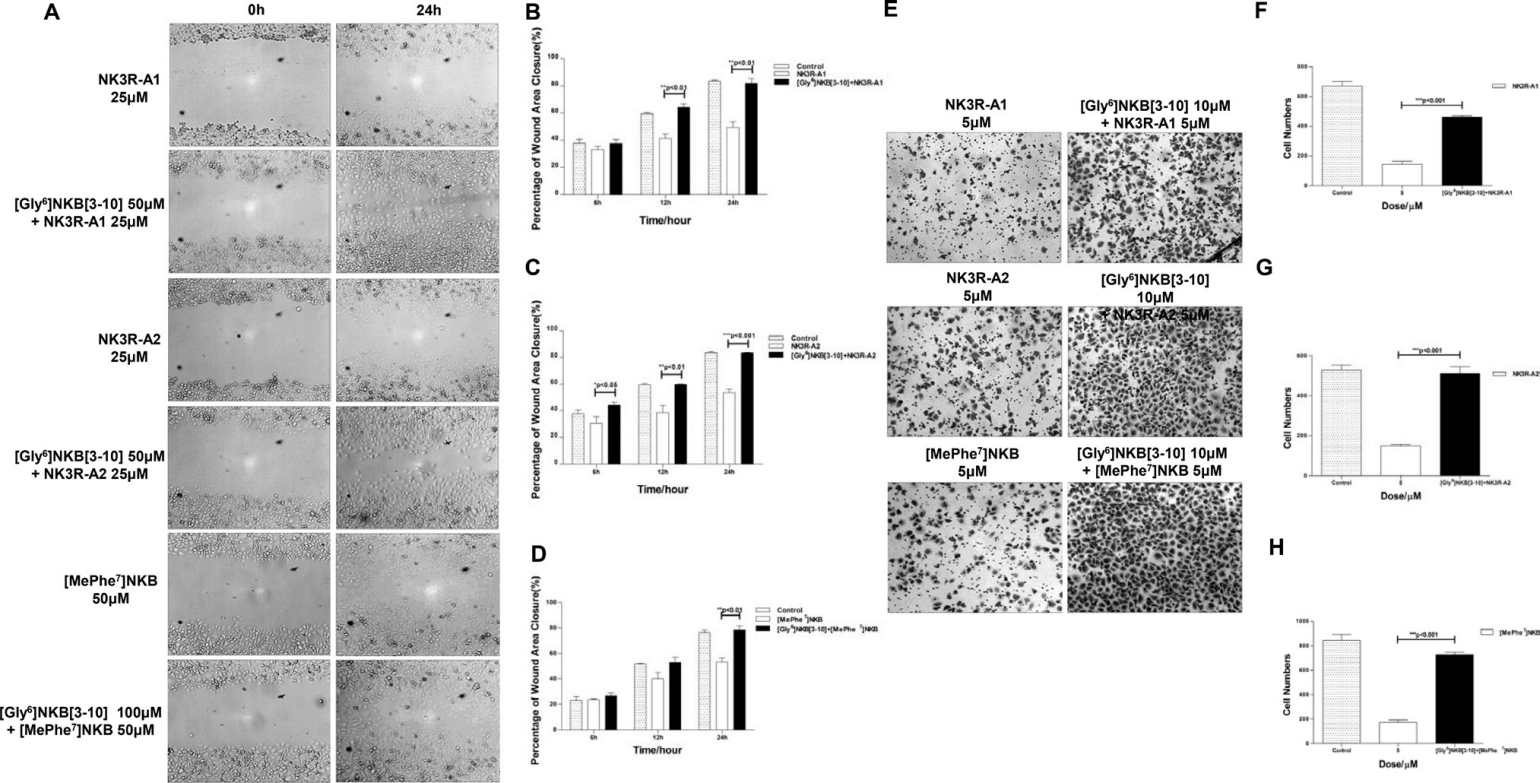
NK3R antagonist [Gly6]NKB[3–10] could antagonize the anti-angiogenic effect induced by NK3R-A1, NK3R-A2 and [MePhe7]NKB *in vitro* (**A**–**D**) Competitive antagonism was measured by using [Gly^6^]NKB[3–10] in Wound healing assay. (A) HUVECs were treated with PBS, NK3R-A1, NK3R-A2 or [MePhe^7^]NKB alone, or co-treated with [Gly^6^]NKB[3–10]. During 24 h healing period, the cells migrating into the wound area were photographed for every 6hand representative photos were shown (200 ×). The analysis of the relative width of wound area was displayed in column chart (**B**–**D**). (**E**–**H**) Competitive antagonism was measured by using [Gly^6^]NKB[3–10] in Transwell migration assay. The representative images are shown (200 ×). (E) The histograms indicated the numbers of migrated HUVECs co-treated with [Gly^6^]NKB[3–10] and NK3R-A1 (F), NK3R-A2 (G) or [MePhe^7^]NKB (H), respectively, in 12 h. Data are mean ± SEM from three independent experiments. *p < 0.05; **p < 0.01; ***p < 0.001.

[Gly^6^]NKB[3–10], specific antagonist of NK3R, was used to further declare the mechanism of the anti-angiogenesis effects of the tested peptides. The closure of the wound areas of cells that treated with [Gly^6^]NKB[3–10] 1 hour before adding NK3R-A1 or NK3R-A2 were nearly complete, reached to ~90% after 24 hours (Figure 4A–4C). Moreover, the anti-migration activity of [MePhe^7^]NKB was also significantly suppressed by [Gly^6^]NKB[3–10] (Figure 4A and 4D).

The antagonist of NK3R countervailed the inhibition effect of all agonist analogues of NK3R, which was able to be further verified in Transwell migration assay. Pretreated with [Gly^6^]NKB[3–10] 1h before adding either NK3R-A1, NK3R-A2 or [MePhe^7^]NKB, the number of migrated cells dramatically increased than that of the [Gly^6^]NKB[3–10] non-treated group (Figure 4E–4H). These findings strongly suggest that NK3R is crucial for the anti-angiogenic activity of the peptides, which means NK3R might be the main target of these testing peptides to drive the function of anti-angiogenesis.

### Antitumor activity of the agonist analogues in S180 sarcoma-bearing BALB/c mice

**Figure 5:**
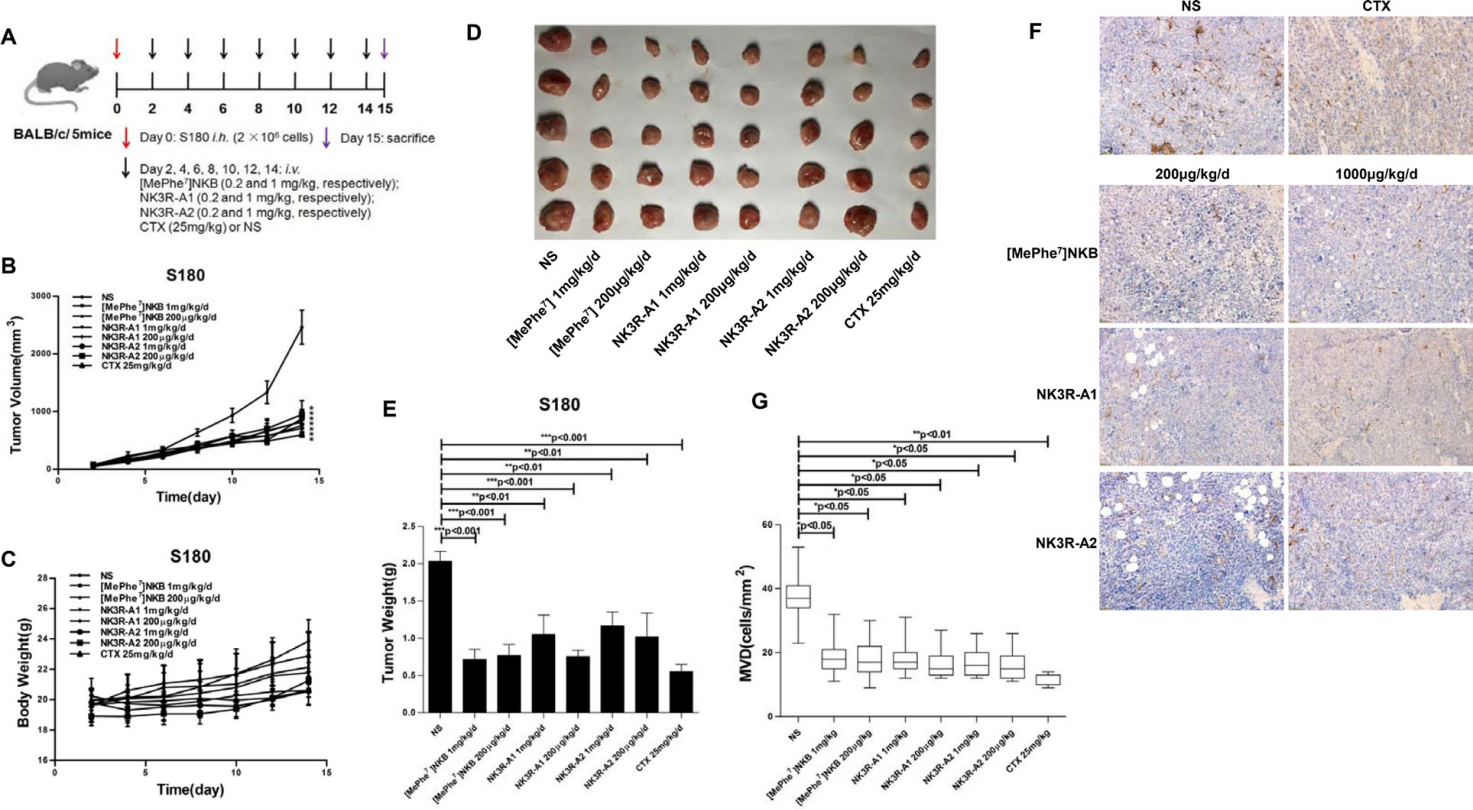
Effects of analogues, NK3R-A1, NK3R-A2 and [MePhe^7^]NKB, on tumor growth in S180 sarcoma-bearing BALB/c mice (**A**) Mice treatment protocol: BALB/c micewere subcutaneously implanted with 2 × 10^6^ cells/mouse on the right flank (day 0). Seventy-two hours after inoculation, forty mice with S180 cells were randomly divided into eight groups. Peptides were continuously administrated intravenously by tails for 14 days (0.1 ml/10 g, once a day): group 1 with NS (negative control), group 2 with cyclophosphamide (CTX, positive control), groups 3 and 4 were injected with peptide [MePhe^7^]NKB (0.2 and 1 mg/kg, respectively), groups 5 and 6 were injected with peptide NK3R-A1 (0.2 and 1 mg/kg, respectively), groups 7 and 8 were injected with peptide NK3R-A2 (0.2 and 1 mg/kg, respectively). All mice were sacrificed after 24 h of the last administration, and the tumor tissues were excised and weighed. (**B**) The growth curves of the tumor were recorded with a caliper and the tumor volume was measured every two day according to the equation: tumor volume = length × width2/2. (**C**) The body weight changes curve of each group of BABL/c mice during the intravenous administration of peptides for 14 days. (**D**–**E**) At the end of the experiment (day 15), the mice were sacrificed, and the tumors were isolated, photographed and weighed. (**F**–**G**) CD31 staining. Tumor tissues from each group were stained using immunohistochemistry for CD31 and representative photos were shown (200 ×) (F), the evaluation of immunostaining for microvasculature density (MVD) in tumor tissues was displayed in bar graph (G). Data are mean ± SEM. *p < 0.05; **p < 0.01; ***p < 0.001.

To characterize the *in vivo* anti-angiogenic activities of the agonist analogues against tumor, S180 sarcoma cells were harvested in BALB/c mice and antitumor properties were analyzed. Experimental results indicated that tumor volume gap between peptides treated and NS control animals progressively increased. And at the end of the experiment, tumor volume of peptides treated groups were over 2 times smaller than those of the control, which illustrated that three agonist analogues of NK3R did counteract the tumor growth in S180 tumor-bearing mice without markedly changes of body weight (Figure 5A–5C). As shown in Figure 5D–5E, the tumor weights of the peptides-treated groups were significantly decreased as compared to the negative control. The IHC staining results (Figure 5F–5G) showed that the microvessel density (MVD) of stained tissues were overtly decreased than that of negative control, which illustrated that the peptides possess anti-angiogenesis activity *in vivo*. In sum, all the results indicated that both NK3R-A1 and NK3R-A2 had potent *in vivo* antitumor effects on S180 sarcoma bearing mice.

## DISCUSSION

In our present work, we proposed a tactics to design analogues of NK3R agonist which has the anti-angiogenesis activity targeting to the blood vessels of tumors. The results demonstrated that the anti-angiogenic activity of three novel peptides, [MePhe^7^]NKB, NK3R-A1 and NK3R-A2, were induced by activating NK3R. Our findings suggested that NK3R might be a novel potential target for the anti-angiogenesis strategy. The anti-angiogenic peptide NK3R-A1 could be used as an template for the development of novel anti-tumor drugs.

Tumor angiogenesis is a complicated process that perturbed the balance between pro-angiogenic and anti-angiogenic mechanisms [21]. During the last three decades, numerous cytokines have been proven to be crucial for tumor related angiogenesis through mediating vasculogenesis, angiogenic remodeling, angiogenic sprouting, and vascular permeability, VEGF is well-known among them [22, 23]. However, with the deepening of the investigations, some limitations of VEGF-related antibodies have been exposed. Bevacizumab in combination with bolus-IFL chemotherapy could increase the risk of suffering gastrointestinal perforation [24]. Ebos JM et al. showed that sunitinib, the VEGFR/PDGFR kinase inhibitor, could accelerate metastatic tumor growth and decrease overall survival in mice after receiving short-term therapy in various metastasis assays [25]. Thus, it is essential to find some novel targets to extend the anti-angiogenic strategy.

NK3R, member of tachykinins receptors, is a class A GPCR preferentially activated by NKB peptide, which together with senktide (a synthetic peptide) are the only known potent and selective agonists of NK3R [26]. Numerous studies indicated that the function of NK3R related to tachykinins exert a plethora of biological effects, including smooth muscle contraction and relaxation, vasodilatation, secretion, activation of the immune system, pain transmission and neurogenic inflammation, and are implicated in a broad range of CNS disorders [27, 28]. Here, we described further evidence that NKB could function as an endogenous angiogenesis inhibitor. Our results also provided novel evidence that the anti-angiogenesis of NKB and its analogues were elicited by activating NK3R, suggesting that NK3R might be a novel potential target for anti-angiogenesis therapeutics.

[MePhe^7^]NKB, another efficient active agonist of NK3R by replacing Val^7^ of NKB with MePhe, has been utilized in our investigation. We demonstrated that [MePhe^7^]NKB which exert even more efficient anti-angiogenic effect compared with NKB on CAM assay and gelatin sponge-CAM assay. And the corresponding competitive assay we performed using the antagonist of NK3R, [Gly^6^]NKB[3–10], showed that it almost completely antagonize the effects of both peptides. In consideration that NKB[4–10], which has been found to be the minimum active sequence of NKB [29], also elicited significant anti-angiogenesis effect in CAM assay (data not shown), [MePhe^7^]NKB[4–10] was therefore singled out for the designation of these two agonist analogues of NK3R, NK3R-A1 and NK3R-A2.

In addition, due to the NK3R is widely distribution, it is necessary to restrict the target selectivity of NK3R and its analogues to the tumor angiogenesis area. Arg-Gly-Asp (RGD) and Asn-Gly-Arg (NGR)are two peptide motifs have been well characterized with known recognition sites among various peptides identified through phage-display to target the tumor vessels of a mouse model. NGR was found to bind the aminopeptidase N (APN/CD13) receptor isoform that is upregulated in tumor vasculature, and shows a three-fold higher specificity than the RGD peptide toward tumor area [19, 20]. In addition, as an endothelial cell-surface receptor of NGR sequence, APN is concentrated on the apical surface of forming blood vessels and is absent or barely detectable in established blood vessels [30]. Colombo and co-workers have confirmed the affinity to its receptor and antitumor activity of linear peptide CNGRC could be lower than that of cyclic peptide c(CNGRC), demonstrating that the disulfide bridge constraint was vital for stabilizing the bent conformation and for increasing the tumor targeting efficiency [31]. With these advantages, the NGR peptide-based drug delivery and imaging studies have been an emerging strategy in anticancer research [32, 33].

To extend the function of NK3R agonists serving as a valid inhibitor of angiogenesis with the selectivity targeting property to the tumor related angiogenesis site, two endothelial cells targeting sequence, NGR and CNGRC, were used to construct novel anti-angiogenic peptide analogues. A GG bridge were utilized to couple the two functional motifs including the tumor vascular recognition sequence NGR or CNGRC, and the anti-angiogenesis sequence of NK3R agonist to impart peptide flexibility and minimize potential steric interactions that would prevent binding [17]. Our results of *in vitro* and CAM assay indicated that the designed two peptides approximately retain the function of [MePhe^7^]NKB and showed even more visible anti-angiogenic effects *in vitro* experiments, which might embody the GG bridge linker maintain the structure of two functional domains. Our results *in vivo* showed that all peptides showed potent antitumor effects without severe toxic effect that which suggested by the finding that the body weight of peptides treated mice did not significantly altered. Meanwhile, NK3R-A1 exert more overt anti-tumor effect than that of NK3R-A2 and [MePhe^7^]NKB, suggesting that agonist analogues of NK3R conjugated with NGR motifs indeed have an advantage over [MePhe^7^]NKB on anti-tumor effects and the disulfide bridge of c(CNGRC) might not be well established, more pharmacokinetics experiments are ongoing.

In conclusion, these results indicated that the complex of NGR or CNGRC and the NK3R agonist are successful for eliciting anti-angiogenic effect. They might open a new gate leading to the anti-angiogenesis targeting therapy. Otherwise, our competition assays also demonstrated the anti-angiogenesis activities of peptides are depending on the activation of NK3R. Our work also proved that NGR-GG-DFF(MeF)GLM- NH_2_ could serve as a novel valued template for the development of anti-angiogenesis drug against cancer.

## MATERIALS AND METHODS

### Peptide synthesis

NKB, [MePhe^7^]NKB, NK3R-A1, NK3R-A2 and the antagonist of NK3R, [Gly^6^]NKB[3–10], were synthesized using a manual peptide solid-phase synthesizer by Fluorenylmethoxycarbonyl (Fmoc) protocol as previously described and the information of the peptides were listed in Table 1. Lyophilized crude peptides were purified by preparative reversed-phase HPLC on a C18 column with an elution gradient of 20–60% acetonitrile with 0.1% trifuoroacetic acid in water. Acquired peptides were confirmed by ESI-MS (KeTai, China).

**Table 1:** Peptide agonists and antagonist for the tachykinin NK3 receptor

Name	Sequence	Mr	References
NKB	Asp-Met-His-Asp-Phe-Phe-Val-Gly-Leu-Met-NH_2_	1211.02	[15]
[MePhe7]NKB	Asp-Met-His-Asp-Phe-Phe-MePhe-Gly-Leu-Met-NH_2_	1272.48	[17]
[Gly_6_]NKB [3–10]	His-Asp-Phe-Gly-Val-Gly-Leu-Met-NH_2_	875.01	[18]
NK3R-A1	Asn-Gly-Arg-Gly-Gly-Asp-Phe-Phe-MePhe-Gly-Leu-Met-NH_2_	1329.0	Novel
NK3R-A2	Cys-Asn-Gly-Arg-Cys-Gly-Gly- Asp-Phe-Phe-MePhe-Gly-Leu-Met-NH_2_	1533.24	Novel

Abbreviation: Relative Molecular Mass, Mr.

### Animals and cell lines

Female BALB/c mice, aged 5–8 weeks (18 ± 2 g), were purchased from Henan Academy of Medical and Pharmaceutical Science (Certificate No. SCXK (Yu) 2011–0010, Henan, China). The animals had free access to food and water in cages that were maintained in a pathogen-free environment (24 ± 0.8°C, humidity of 55 ± 5%) with a 12 hours light/dark cycle. All animal experimental procedures were approved by Zhengzhou University committee for animal experiments. Human umbilical vein endothelial cells (HUVECs) and murine sarcoma S180 cell line were maintained in our laboratory. All cells were cultured in RPMI 1640 (Invitrogen, USA) with 10% fetal bovine serum, 100 U/ml penicillin, and 100 μg/ml streptomycin at 37°C in a humidified atmosphere of 5% CO_2_.

### Cell viability assay

HUVECs were used to evaluate the effects of different concentrations of peptides [MePhe^7^]NKB, NK3R-A1 and NK3R-A2. The effects of peptides (5, 25, 100 μM) on cell proliferation were determined by a quantitative colorimetric assay with 3-[4,5-dimethylthiazol-2-yl]-2,5-diphenyl-tetrazolium bromide (MTT).

### Wound healing assay

Wound healing assay was performed to validate the anti-angiogenic effect of peptides *in vitro* and investigate the relevant mechanism. 50 μM of NK3 receptor antagonist, [Gly^6^]NKB[3–10], was administrated 1 hour before adding agonists. Images of migrated cells were taken using an inverted microscope at 100 × magnification after every 6h of incubation in a humidified atmosphere with 5% CO_2_ at 37°C. The pictures were taken from three randomly selected fields and analyzed by Image J.

### Transwell migration assay

The Transwell migration assay is a commonly used test to assess the migratory response of endothelial cells to angiogenic inducers or inhibitors. The effect of peptides [MePhe^7^]NKB, NK3R-A1 and NK3R-A2 (1, 5, 25, 100 μM) on HUVECs migration was performed using a Transwell system (BD, USA) with pore size of 8.0 μm as described previously [34]. Five random views of successfully migrated cells were photographed and quantified under an inverted microscope. The competitive antagonism assay was conducted by using [Gly^6^]NKB[3–10].

### Chick chorioallantoic membrane (CAM) assay

The CAM assay was carried out as described with slight modifications according to the procedure described by Le Noble FA et al previously [35]. 1 μM of [Gly^6^]NKB[3–10] were added to eggs 1 hour before adding the agonist to validate the anti-angiogenic role of peptides. Pictures were disposed with picture soft (NIS-Elements Basic Research, Nikon Corporation, Japan). The anti-angiogenesis effect was calculated using the following equation: [( Vessel Number of CAM treated by drug/ (Vessel number of CAM treated by PBS)] ×100% = % relative rate of vascular density. [(Vessel area of CAM treated by drug/(Vessel area of CAM treated by PBS)] ×100% = % relative rate of vascular area.

### The gelatin sponge –CAM assay

The gelatin sponge-CAM assay was performed as previously described by Ritabbi D [36]. Briefly, sterilized gelatin sponges (Fukang Medical Devices Co. Ltd, Guilin, China) containing peptides were implanted onto CAM on day 8 of incubation. Embryos and membranes were fixed and cut *in ovo* after 48 h treated with peptides. Samples were sent to Servicebio Technology Company (Wuhan, China) to conduct special staining. The stained CAM tissues were observed by an inverted microscope (400 ×).

### Mice Treatment

The bearing mice model of S180 cells were performed following the protocol described previously [37]. Mice were injected (i.h.) with 2 × 10^6^ S180 cells at day 0. 24 hours later, [MePhe^7^]NKB, NK3R-A1 and NK3R-A2 (0.2 or 1 mg/kg/d) was administered by the intravenous (i.v.) route for 14 days. Mice were administered Normal Saline (NS) or CTX (25 mg/kg/d; Sigma-Aldrich, Rome, Italy) as negative control and positive control, respectively. Positive control group were i.v. administered 25 mg/kg/d for two weeks. The tumor volumes were monitored every two days, and primary tumor volumes were calculated by the formula V = a × b^2^/2, where “a” is the longest dimension parallel to the skin surface and “b” is the dimension perpendicular to “a” and parallel to the surface. All mice were sacrificed after 24 hours of the last administration, and the tumor tissues were excised and weighed.

### Immunohistochemical staining

The anti-CD31 antibody (bs-0195R) was purchased from Bioss Company (Beijing, China) and used according to the manufacturer's instructions. Tumor tissues were sent to pathology of Zhengzhou Yihe hospital to stain. The stained sample tissues were observed under an inverted microscope (200 ×) using the method of Weidner [38].

### Statistical analysis

Data were presented as mean ± SEM. The statistical significances of difference between each group were analyzed by one-way analysis of variance (ANOVA). Statistical differences were presented at probability levels of *p < 0.05, **p < 0.01, and ***p < 0.001.
